# Area Disease Estimation Based on Sentinel Hospital Records

**DOI:** 10.1371/journal.pone.0023428

**Published:** 2011-08-23

**Authors:** Jin-Feng Wang, Ben Y. Reis, Mao-Gui Hu, George Christakos, Wei-Zhong Yang, Qiao Sun, Zhong-Jie Li, Xiao-Zhou Li, Sheng-Jie Lai, Hong-Yan Chen, Dao-Chen Wang

**Affiliations:** 1 State Key Laboratory of Resources and Environmental Information System, Institute of Geographic Sciences and Natural Resources Research, Chinese Academy of Sciences, Beijing, China; 2 Harvard Medical School, Children's Hospital Boston, Boston, Massachusetts, United States of America; 3 Department of Geography, San Diego State University, San Diego, California, United States of America; 4 Office for Disease Control and Emergency Response, Chinese Center for Disease Control and Prevention (CDC), Beijing, China; 5 Shanghai Pudong New Area Center for Disease Control and Prevention, Shanghai, China; 6 School of Environmental Sciences, University of Liverpool, Liverpool, United Kingdom; 7 MGS, Manchester, United Kingdom; Fred Hutchinson Cancer Research Center, United States of America

## Abstract

**Background:**

Population health attributes (such as disease incidence and prevalence) are often estimated using sentinel hospital records, which are subject to multiple sources of uncertainty. When applied to these health attributes, commonly used biased estimation techniques can lead to false conclusions and ineffective disease intervention and control. Although some estimators can account for measurement error (in the form of white noise, usually after de-trending), most mainstream health statistics techniques cannot generate unbiased and minimum error variance estimates when the available data are biased.

**Methods and Findings:**

A new technique, called the Biased Sample Hospital-based Area Disease Estimation (B-SHADE), is introduced that generates space-time population disease estimates using biased hospital records. The effectiveness of the technique is empirically evaluated in terms of hospital records of disease incidence (for hand-foot-mouth disease and fever syndrome cases) in Shanghai (China) during a two-year period. The B-SHADE technique uses a weighted summation of sentinel hospital records to derive unbiased and minimum error variance estimates of area incidence. The calculation of these weights is the outcome of a process that combines: the available space-time information; a rigorous assessment of both, the horizontal relationships between hospital records and the vertical links between each hospital's records and the overall disease situation in the region. In this way, the representativeness of the sentinel hospital records was improved, the possible biases of these records were corrected, and the generated area incidence estimates were best linear unbiased estimates (BLUE). Using the same hospital records, the performance of the B-SHADE technique was compared against two mainstream estimators.

**Conclusions:**

The B-SHADE technique involves a hospital network-based model that blends the optimal estimation features of the Block Kriging method and the sample bias correction efficiency of the ratio estimator method. In this way, B-SHADE can overcome the limitations of both methods: Block Kriging's inadequacy concerning the correction of sample bias and spatial clustering; and the ratio estimator's limitation as regards error minimization. The generality of the B-SHADE technique is further demonstrated by the fact that it reduces to Block Kriging in the case of unbiased samples; to ratio estimator if there is no correlation between hospitals; and to simple statistic if the hospital records are neither biased nor space-time correlated. In addition to the theoretical advantages of the B-SHADE technique over the two other methods above, two real world case studies (hand-foot-mouth disease and fever syndrome cases) demonstrated its empirical superiority, as well.

## Introduction

Important disease attributes include incidence, prevalence and mortality [1], [2]. For example, the adequate understanding of incidence distribution across space-time plays a key role in disease causation, pattern description, trend prediction, outbreak identification, medical resources allocation, and drug treatment assessment [3], [4], [5], [6], [7]. Exhaustive population surveys are often too expensive and time consuming, especially when timeliness is a critical issue in infectious disease control. As a result, the incidence distribution is often estimated with the help of sentinel hospital-based surveillances. Conventional and spatial sampling techniques [8], [9] are well established in a probability setting. The World Health Organization (WHO), for example, has published a comprehensive sampling design manual [10] based on simple random sampling. Although it is relatively easy to use them, the manual's techniques have exhibited low sampling efficiency [9]. Mainstream statistical sampling methods used in epidemiology, frequently assume randomly collected samples at the global or the local scales [10]. However, the estimation of a population health attribute (and its confidence limits) is strongly affected by sample selection and the statistical technique implemented. For sampling purposes, sentinel hospitals can be chosen randomly, systematically, or by stratified analysis, either in the attribute or in the spatial domains [8], [9]. Area population incidence estimates are then generated from the spatial sample using a statistical technique [8], [9], [10], [11]. However, these sample estimates can be biased if the randomness assumption is not valid in real world situations, in which case the expected estimation error is known as bias [12].

Sentinel hospital-based incidence estimates are often biased, for several reasons. First, hospitals with advanced electronic management systems (which allow quick and high quality reporting) are much more likely to be selected as sentinel for data “cherry-picking” purposes [13]. Second, poor management or a hospital's lack of capacity may cause significant underreporting [3]. Third, the sampling strata approach may not reflect major population variation features [11]. For example, HBsAg (Hepatitis B surface antigen) prevalence is often surveyed based on geographical stratification, even though prevalence of this disease is strongly stratified by age in China. This mismatch results in biased prevalence estimation. Other sources of bias in epidemic studies may be due to classification and measurement inaccuracies, and unfair comparisons. For example, the reliability of verbal autopsy (which assigns a probable cause of death based on interviews with families regarding the deceased's symptoms) may include a built-in bias [4]. Correction of sample selection bias in the case of linear regression has been extensively studied in econometrics [5], and more recently in terms of machine learning algorithms. Furthermore, a number of epidemic studies have explicitly dealt with bias issues [6], [14]. The most common techniques of bias correction involve assumptions about missing probability, training error reweighting [5], or using empirical sample-population ratios [6], [14]. Network connections between hospitals and spatial dependence models have the potential to further improve surveillance efficiency [9], [15], [16].

In this paper we introduce a new technique, called the B-SHADE (Biased Sentinel Hospital based Area Disease Estimation) technique, which generates best linear unbiased estimates (BLUE) of health attributes using biased hospital samples. Note that the B-SHADE technique can be used to study any health attribute that varies across space-time. This technique incorporates the “vertical” relationship of ratios between hospital samples and the overall disease incidence to treat sampling bias, as well as the “horizontal” correlation between all hospitals to increase the efficient sample size and reduce estimation variance. The structured network model takes into account the links between data streams, in addition to isolated streams. Thus, B-SHADE is a model-assisted and data-driven technique, which corrects sample bias by means of a data adaptive process using fewer assumptions and without requiring a deeper knowledge of the bias mechanism. Empirical examples will be considered below that provide valuable insight and demonstrate the merits of the new technique.

## Methods

The objective of the present study is to estimate the number of disease cases in an area based on incidence reports from sentinel hospitals. In particular, the actual number of cases in the entire area per time unit (weekly, say) is given in theory by
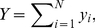
(1)where the hospital sample includes *n* sentinel hospitals out of a total number of 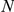 (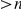) hospitals in the area; and 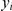 is the weekly number of cases reported by the *i*-th hospital. The actual *Y* must be estimated from the available sentinel hospital records (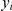, 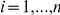). These reports are properly weighted to correct for possible bias, the fact that 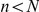, and also to take into account correlations among all hospitals (which can increase the effective sample size), as follows,
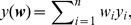
(2)where *w_i_* denotes the weight (contribution) of the *i*-th sentinel hospital report to the incidence estimate of interest. Otherwise said, the *y*(***w***) in Eq. (2) is an estimate of the actual (but unknown) *Y* in Eq. (1). As should be expected, the two properties of the incidence estimate of Eq. (2) are: unbiasedness, i.e. *E*(*y*(***w***)) = *E*(*Y*), where *E* denotes statistical expectation; and minimum estimation variance, i.e. 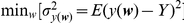.

### Associations between Hospitals

**Figure 1:**
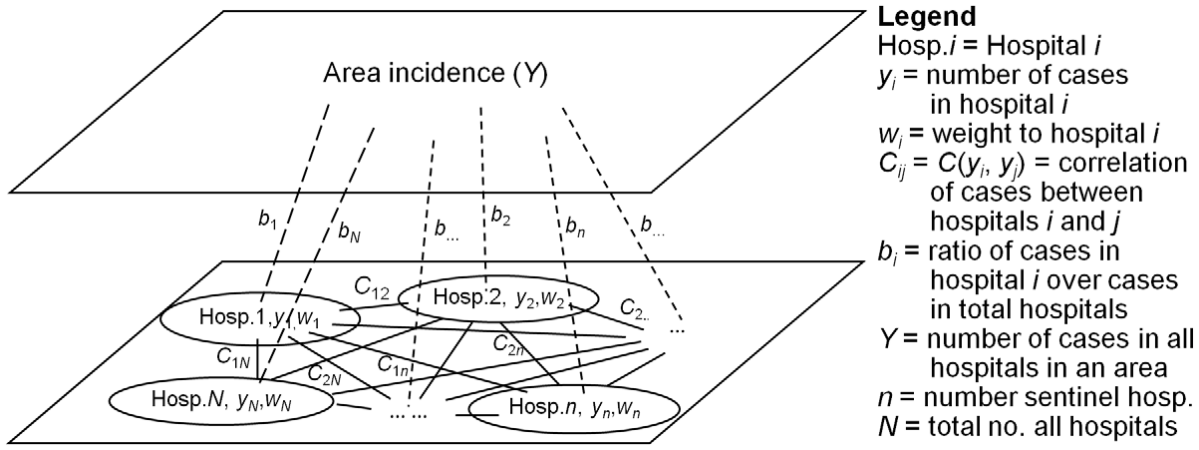
Relationship between hospitals and relationship between hospitals and area incidence.

The number of disease cases reported by the hospitals is one of the most important inputs of incidence estimation during routine surveillance and in case of a disease outbreak. This population health characteristic is usually estimated in terms of the (direct or indirect) links between regional population and sentinel hospitals. Underlying these links is an intricate social network of individual and hospital across the region of interest. In Figure 1, *b_i_* is the ratio between the sample incidence (*y_i_*) and the population incidence (*Y*); and *C_ij_* denotes the dependency of disease records *y_i_* and *y_j_* in the hospitals *i* and *j*, respectively, as measured by the covariance function between the incidences at hospital pairs, i.e. *C_ij_* = *C*(*y_i_*, *y_j_*). The relationships between hospital records (solid lines in Figure 1), as well as between sentinel hospital records and the total number of disease cases in the study area (dashed lines in Figure 1) are taken into account.

### Systematic Modelling

As mentioned earlier, an objective of the current study is to derive incidence BLUEs across space-time based on biased sentinel hospital records. This objective is similar to that of Block Kriging that generates BLUEs for spatially varying phenomena [11], [15], [17]. Block Kriging represents the disease distribution as a random field [15] with spatially homogeneous covariances that are functions of the spatial distance (i.e., the spatial metric assumed is Euclidean). However, these assumptions are not satisfied in the case of sentinel hospital records. Similar is the case of spatial regression techniques [18]. Indeed, the hospitals are discretely distributed in space and it may not be appropriate to assume that the disease cases reported by them are spatially homogeneous in the continuous random field sense. Moreover, the sentinel hospital records are often subject to sample selection bias; and spatial covariances between hospitals are not necessarily functions of the distance between them (i.e., the metric maybe non-Euclidean). In view of the above considerations, the B-SHADE technique assumes an empirical non-Euclidean metric that is appropriate for the specific study objectives, and it can correct for sample bias.

As we saw earlier, the number of disease cases *Y* in an area is estimated in terms of the weighted sample total *y*(***w***) that satisfies two conditions: it is an unbiased estimate of *Y*, and it minimizes the mean squared estimation error. The first condition implies that (File S1)
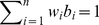
(3)where 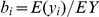 represents the bias of hospital *i* with respect to the total number of hospitals, and the weight 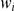 expresses the contribution of each hospital *i* to disease estimation. The second condition implies that these weights are calculated by minimizing the estimation variance of Eqs. (1)–(2), i.e.
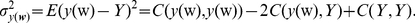
(4)Minimizing 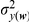 with respect to the weights 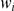 (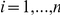) and taking into account the unbiasedness Eq. (3) gives,
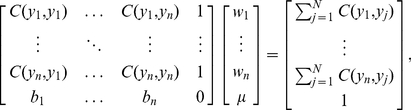
(5)where 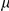 is a Langrange multiplier (File S1). The minimized estimation error variance can be then written as,
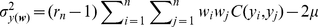
(6)where 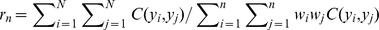 is the ratio of the summation of correlations among all hospital records over the summation of the sample-based estimates. Actually, the 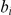
's and 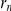 express the contributions of the 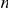 sentinel hospitals (relative to the total number 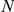 of hospitals) to the incidence mean and incidence correlation, respectively. The empirically significant parameters 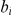 and 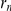 can be computed using temporal observations. The part 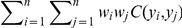 of Eq. (6) expresses sentinel hospital associations across space computed in real time. The 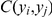, *b_i_* and *r_n_* can be estimated empirically from historical data.

In sum, the area incidence estimation procedure is as follows: (*a*) The covariance values *C*(*y_i_*, *y_j_*) are calculated from the sentinel hospital records (see, also, Figure 1). (*b*) Given these values, Eqs. (5) are solved with respect to 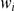 (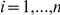) and 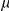. (*c*) The calculated 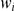 and 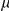 are substituted in Eqs. (2) and (5) to find the desired incidence estimate and the associated estimation error variance, respectively.

## Results

### Study Region

**Figure 2:**
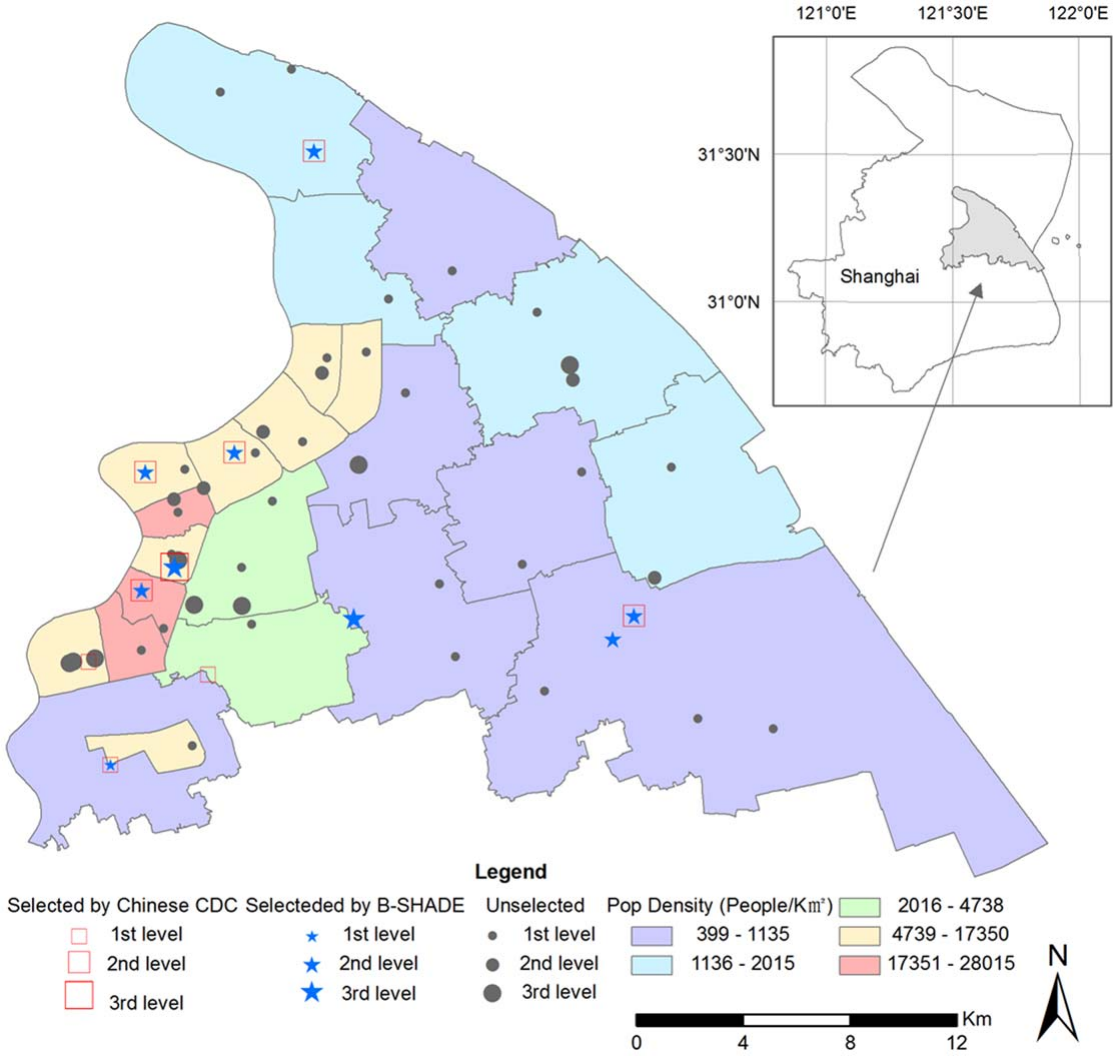
Study region and sentinel hospitals.

Two cases will be used to compare the B-SHADE's performance in Pudong District of Shanghai. The Shanghai 2010 World Expo took place in the Pudong District of Shanghai (China), from April 30 through October 30, 2010. The Chinese Center for Disease Control and Prevention (CDC) and the Pudong CDC were responsible for health surveillance in the surrounding area during this major event. Daily incidences of hand-foot-mouth disease (HFMD) at all 53 hospitals were collected in the Pudong District from January 1, 2009 to September 9, 2010. These data are used in the present study to evaluate the performance of the B-SHADE technique vs. other methods. Figure 2 shows the locations and levels of the 53 hospitals in the District, and also indicates the 9 sentinel hospitals recommended by the Chinese CDC, as well as the 9 sentinel hospitals chosen by the B-SHADE technique.

### Algorithm

**Figure 3:**
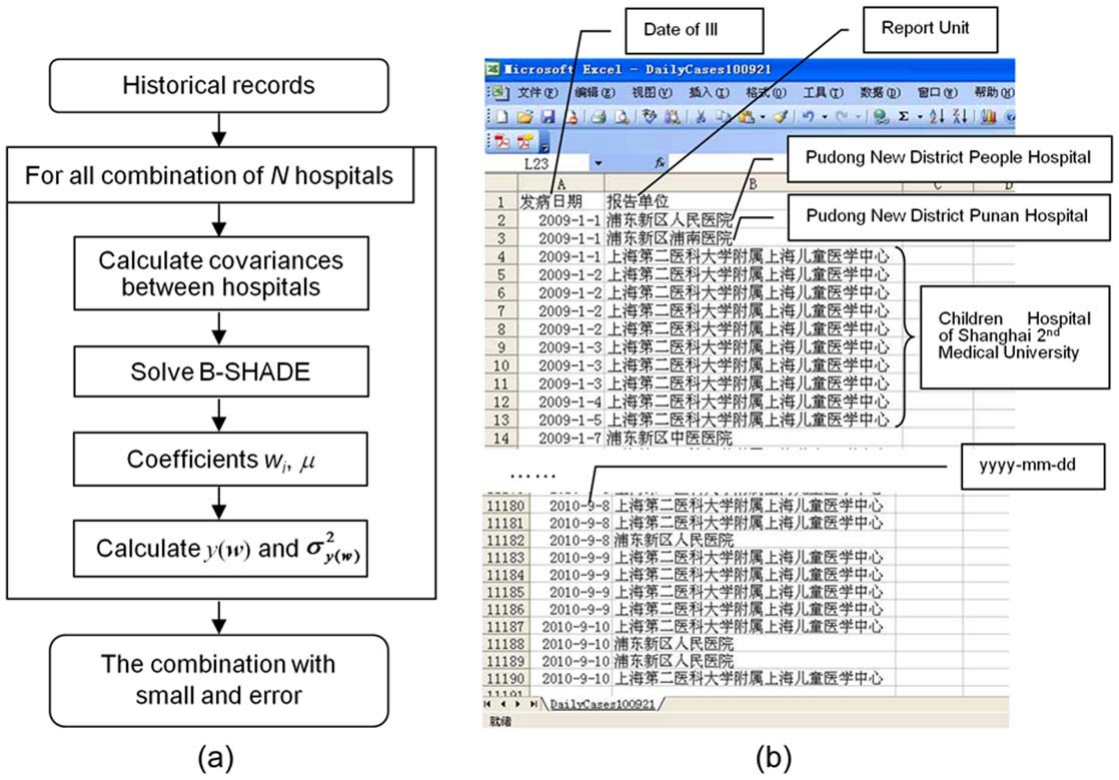
Flowchart of selected sentinel hospitals using B-SHADE. (a) B-SHADE algorithm; (b) Historical records as B-SHADE input.

The flowchart of B-SHADE estimation is shown in Figure 3a. The historical weekly incidence records (Figure 3b) are used as repeated samples to compute the *b_i_* coefficients and the covariances *C_ij_* = *C*(*y_i_*, *y_j_*). The *b_i_* is assigned to hospital *i*, and *C_ij_* links the records at the hospitals *i* and *j*. Both *b_i_* and *C_ij_* are metric (distance)-independent. Given the required number *n* of sentinel hospitals (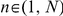), we list all combinations of *n* out of a total of *N* = 53 hospitals in the Pudong District, Shanghai. For each one of the *C_N_^n^* combinations, the weights 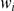 and *μ* were computed in terms of Eq. (5). In practice, an optimization scheme is usually adopted to accelerate the speed of calculation convergence [19]. Subsequently, the estimated total number of cases *y*(***w***) and the associated estimation error variance 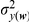 were computed by means of Eqs. (2) and (6). Among all outcomes of the *C_N_^n^* combinations, that with the smallest error variance and absolute estimation error was selected as the best sentinel hospital choice for the given number *n*. For illustration, Figure 3b gives a sample of data input (historical records) for the B-SHADE technique.

It is noteworthy that the *C_ij_* between all hospitals are estimated using the historical weekly records between all hospitals rather than by fitting a standard covariance model (as is done, e.g., in Block Kriging). Otherwise said, B-SHADE is a network-based technique that does not depend on the form of the Euclidean distance, as is generally the case with the regression family of techniques [15]. Covariance calculations can be improved if the dependence structure of disease incidences between consecutive time intervals is taken in to account. The above goals can be achieved by means of the Kalman filtering and the BME techniques [1], [15], [20].

### Performance

The B-SHADE technique was compared with two other commonly used techniques, as follows:

1. B-SHADE technique, *y*(***w***) (*t*), see File S1;
2. Ratio estimator technique, 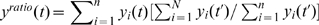, see File S2;
3. Simple random estimator technique, 
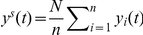
, see File S3.

Let *y^est^*(*t*) denote the estimated number of disease cases during week *t*. Herein, for convenience the *y^est^* may denote *y*(***w***), *y^s^* or *y^ratio^*. The performance of these three techniques is measured by means of the absolute error
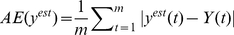
(7)where *m* is the number of weeks considered. In the case of a new sampling project, the *Y* may be unknown, in which case the precision of an estimate *y^est^* is measured by means of the error variance of Eq. (6), which is an interesting property that B-SHADE shares with Block Kriging.

**Figure 4:**
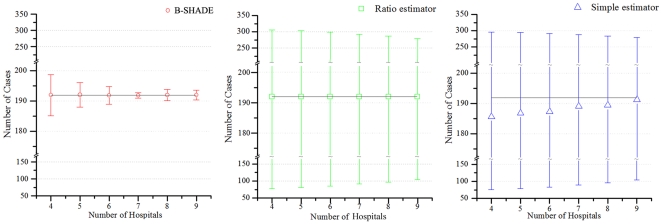
Average number of cases and associated standard deviation estimated by the three estimators.

**Figure 5:**
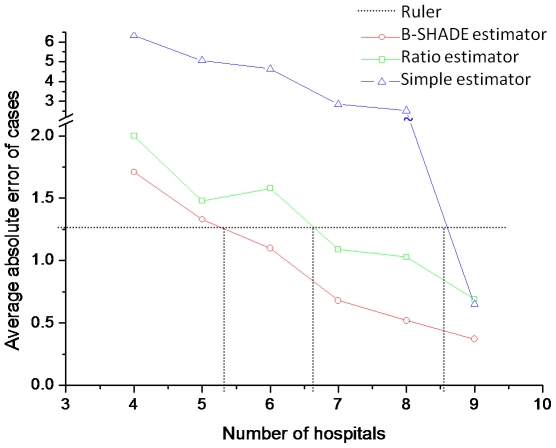
Average absolute error of weekly Hand-Foot-Mouth Disease cases estimated by the three estimators.

As already mentioned, the B-SHADE technique provides BLUEs of area disease incidences. Table 1 summarizes the average absolute error (*AE*) weekly cases estimated by the three techniques above using 9 hospitals during the period of the 3^rd^–34^th^ weeks of 2010. The 9 sentinel hospitals are the Shanghai East Hospital, Gongli Hospital, Punan Hospital, People's Hospital, Sanlin Community health service center, Pudong Hospital of Traditional Chinese Medicine, Shanghai Children's Medical Center, Shanghai Seventh People's Hospital, and Shuguang Hospital, Pudong (Figure 2). Similarly, Figure 4 shows the average number of weekly cases and the corresponding standard deviations estimated by the three techniques. Figure 5 displays the average absolute estimation error of weekly cases obtained by the three techniques using the same set of sentinel hospitals records during the same time period. Also, the B-SHADE technique consistently exhibits the smallest estimation error variance among all three techniques. Table 1 clearly shows that,
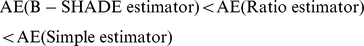
The B-SHADE technique outperforms the other two techniques in the empirical example considered above. In Figure 4 the average numbers of disease cases obtained by the three techniques seem to be close to each other, but the B-SHADE exhibits significant smaller statistical standard deviations. B-SHADE uses a smaller number of samples than the other two techniques in order to meet the required precision (this is illustrated in Table 1 and Figure 5). For example, if one draws a horizontal line at 1.25*AE* (Figure 5), it can be seen that 5, 6 and 8 sentinel hospitals are needed by the B-SHADE, ratio and simple estimators, respectively, in order to meet the required precision.

**Table 1:** Average Absolute Error of Weekly Hand-Foot-Mouth Disease Cases Estimated by the Three Estimators.

Number of hospitals	B-SHADE	Ratio estimator	Simple estimator
2	8.64	10.63	49.24
3	5.89	10.65	48.38
4	1.71	2.00	6.32
5	1.33	1.48	5.06
6	1.10	1.58	4.65
7	0.68	1.09	2.85
8	0.52	1.03	2.53
9	0.37	0.69	0.65

### The Fever Syndrome Case Study

The B-SHADE technique was also applied to another real world case study, namely, the space-time estimation of fever syndrome cases in the same study region as above, from April 30 to October 28, 2010. The actual total number of fever syndrome cases during this period was more than 50,000 as reported by 35 hospitals. To reduce computation time, 9 hospitals (with case numbers less than 50 during the 26-weeks period) were removed. The total number of fever syndrome cases at these 9 hospitals was 175. Among the remaining 26 hospitals, 18 of them were selected as sentinel hospitals by the Shanghai CDC. The fever syndrome cases during the first 16 weeks were selected. These data were then used by the B-SHADE technique to estimate the total case numbers. The average absolute errors of the derived disease estimates for the 3, 6,…,18 sentinel hospitals (listed in Table 2) show that the B-SHADE technique performed well in this case study too.

**Table 2:** Average Absolute Error of Weekly Fever Syndrome Cases Estimated by the Three Estimators.

Number of hospitals	B-SHADE	Ratio estimator	Simple estimator
3	499	6422	713
6	247	5258	275
9	160	3169	182
12	121	1830	143
15	119	1130	167
18	88	641	122

## Discussion

Historically, Snow's work in 1855 [21] was the first study to clearly show that public health measures can have enormous contributions to population health. In addition to its significance in population health risk assessment, the information obtained by statistical epidemiology could be used to improve clinical practice. For example, the natural history of an individual's health state, the most important element in evidence-based medicine [22], are estimated in terms of the more stable states of stratified population. Individual's health risk may be judged by means of the prevalence and incidence of populations with similar characteristics, from a spatiotemporal synthesis perspective [23].

Important health assessment attributes, like area disease incidence, can be estimated from biased sentinel hospital records. A simple random estimator technique is frequently used in practice, which is BLUE only if the population health distribution is independent and identically distributed (iid) [8], [24]. For spatially correlated phenomena, the Block Kriging technique [12] capitalizes on spatial dependences to estimate population incidences in terms of weighting coefficients that minimize the estimation error variance. However, the BLUE feature of Block Kriging could be inadequate if its spatial homogeneity assumption is not realistic and the samples are biased. The ratio estimator technique can be used to derive unbiased estimates using a biased sample [6], [14], but the technique is unable to handle estimation errors.

In sum, the above techniques have different limitations when applied to population health assessment. These two techniques have been blended by the B-SHADE technique in a way that their relative limitations are eliminated. In the B-SHADE setting, the efficient sample size is improved by taking into account spatial correlations between hospitals. The coefficients *b_i_* offer a simple way to correct the bias of sentinel hospital records. Observation models of the *b_i_* with varying levels of sophistication may be constructed. A fact contributing to its generality is that the B-SHADE technique reduces to Block Kriging if the *b_i_* is the same for all hospitals; it reduces to the ratio estimator technique if there are no horizontal correlations between hospitals; and it reduces to the simple random estimator technique when the between hospitals correlation is weak and the sample is unbiased.

In addition to the two useful B-SHADE features above (namely, the BLUE property and the correction of sample bias), as a result of its mathematical formulation the technique has two more important properties: resistance to spatial clustering of sentinel hospitals and data stream surge. In epidemiology, population surges have been found to affect multiple data streams in a similar manner, but the relationships among the various data streams are not significantly affected [25]. Thus, the population interaction network and its parameters are rationally assumed to be stable over time and are estimated based on previous records. B-SHADE is a hospital network-based technique that outperforms historical models especially since it can fluctuate with the unpredictable incidence shifts (e.g., population surges during big events such as the Olympics and the World Expo [16]) by rigorously modelling the interactions between the hospitals and real time samples. This technique also outperforms traditional spatial models. For example, the B-SHADE technique avoids superficial geometrical clustering of sample data because it depends on the hospital network rather than on the Euclidean distance between hospitals.

Two different real world applications involving the Shanghai World Expo 2010 showed that the B-SHADE technique can be easily applied to real time disease surveillance, and that it works well in the sense of producing small absolute errors. The technique can be also used to select the best sentinel hospitals for the specified area incidence BLUEs purposes. The precision of the B-SHADE technique relies on the strong and stable relationships as well as the spatial correlations between hospitals, which are adequately represented by the theoretical structure of the technique and its empirical parameters.

Vertical representations and horizontal correlations between hospitals (in terms of *b_i_* and *C_ij_*, respectively) together with the BLUE objective (expressed by 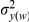) make up the framework of network-based statistics. Under the assumption that the hospital network characteristics at the present time are similar to those at other times in the network's history, the parameters of the B-SHADE technique are estimated using historical data. When the hospitals have long enough records (say, 10 or more), they are used as repeated samples for parameter estimation purposes. If only a fraction of the hospitals have historical data or the datasets are not long enough, the estimation of *b_i_* and *C_ij_* for all hospitals needs further investigation.

Unbiasedness is a statistical property that is usually sought when estimating a disease attribute or designing a sampling scheme [5], [8], [10]. This is also the case with the B-SHADE technique. The Kalman filter technique seeks a minimum variance estimate, without this being necessarily an unbiased one [15]. Precision losses due to unbiasedness and their implication in epidemic studies deserve further investigation.

While the ratio estimator and the B-SHADE techniques treat sample bias by means of the coefficients *b_i_* (regardless of the bias causes), the technique suggested by Hechman (1979) handles selection bias in terms of two equations: one models observations and another one models the selection mechanism, aiming at reaching an unbiased estimate (although not a BLUE one). The Hechman estimator is a good option if the bias mechanism is known and the historical dataset is not rich. Hierarchical Bayesian (HB) modelling [9] may handle the sample bias by setting the bias rate as latent variable with a probability distribution. When probability distributions assume statistical independence, it is necessary to first modify the distributions to incorporate correlations between hospitals, and then HB is applied to the correlated phenomena. On the other hand, the BME technique [1], [15], [23], [26] considers unbiasedness in a knowledge synthesis framework, and shows that it is a space-time estimation property that may depend on the substantives features of the real world situation. So, in some cases the estimate that on average is equal with the actual mean is appropriate (unbiasedness), whereas in some other cases biasedness maybe desirable in order to include valuable prior information in the space-time estimation process. The above are issues that deserve further investigation.

### Supporting Information

The readers are referred to the detailed flowchart of Figure 3 in order to develop their own B-SHADE algorithm and apply it to the dataset of their interest. The readers can contact us for any assistance they may need in the process of developing and applying a B-SHADE algorithm (wangjf@igsnrr.ac.cn or humg@lreis.ac.cn). We expect that an interactive B-SHADE package will be released on the Internet in the near future for public use (free of charge).

## Supporting Information

File S1 **Derivation of the main B-SHADE equations.**
(DOC)

File S2 **A brief review of the ratio estimator.**
(DOC)

File S3 **A brief review of the simple random estimator.**
(DOC)
