# Supplementary material for: Area Disease Estimation Based on Sentinel Hospital Records
Source: PLoS One. 2011 Aug 23;6(8):e23428. doi: 10.1371/journal.pone.0023428 (PMC3160318; doi:10.1371/journal.pone.0023428)
Supplement: File S1 — Derivation of the main B-SHADE equations. (DOC) [file pone.0023428.s001.doc]

**Supporting Information File 1. Derivation of the main B-SHADE equations.**

As above, let *y_i_* be the number of disease cases reported by hospital *i*, and let *Y* of Eq. (1) be the observed number of cases reported by all *N* hospitals in the area during a time unit (say, a week), *n* denotes the number of sentinel hospitals. One can estimate *Y* by the weighted sum of the sentinel hospital cases, i.e., Eq. (2). The *y*(***w***) satisfies two conditions: (a) it is an unbiased estimate of the observed total population cases *Y*, and (b) it minimizes the mean squared estimation error (MSEE), .

The first condition implies that,, or $\sum_{i=1}^{n} w_{i} Ey_{i}/EY=1$, which leads to Eq. (3). Concerning the second condition, the MSEE is given by: $E((y(w)-Y)-E(y(w)-Y))^{2}=C(y(w),y(w))-2C(y(w),Y)+C(Y,Y)$, i.e. Eq. (4). The 1st term in the right of Eq. (4) is

, or

(A1)

The 2nd term in the right of Eq. (4) is $2C(y(w),Y)=2E(y(w)-Ey(w))(Y-EY)$
$=2(EYy(w)-Ey(w)EY)$
$=2\sum_{i=1}^{n} w_{i}(Ey_{i}Y-Ey_{i}EY)=2\sum_{i=1}^{n} w_{i}\sum_{j=1}^{N} (Ey_{i}y_{j}-Ey_{i}Ey_{j})$, or

. (A2)

And the 3rd item is $C(Y,Y)=E(Y-EY)^{2}=E(\sum_{i=1}^{N} (y_{i}-Ey_{i}))^{2}=\sum_{i=1}^{N} \sum_{j=1}^{N} E(y_{i}-Ey_{i})(y_{j}-Ey_{j})$, or

$C(Y,Y)=\sum_{i=1}^{N} \sum_{j=1}^{N} C(y_{i},y_{j})$ (A3)

By substituting Eqs. (A1)-(A3) into Eq. (4) one finds,

$\sigma_{y^{w}}^{2}=\sum_{i=1}^{n} \sum_{j=1}^{n} w_{i}w_{j}C(y_{i},y_{j})-2\sum_{i=1}^{N} \sum_{j=1}^{n} w_{j}C(y_{i},y_{j})+\sum_{i=1}^{N} \sum_{j=1}^{N} C(y_{i},y_{j})$. (A4)

To minimize Eq. (A4) subject to the unbiasedness condition of Eq. (3) is a standard constrained optimization problem [15] that leads to the minimization of the quantity , where $\mu$ is a Lagrange multiplier. Next, the partial derivatives of $L_{y(w)}$ wrt to $w_{i}$ and $\mu$ are set equal to zero. The  gives the unbiasedness condition of Eq. (3). Furthermore, , or , or , or $2\sum_{j=1}^{n} w_{j}C(y_{i},y_{j})-2\sum_{j=1}^{N} C(y_{i},y_{j})+2\mu b_{i}=0$, or

$\sum_{j=1}^{n} w_{j}C(y_{i},y_{j})+\mu b_{i}=\sum_{j=1}^{N} C(y_{i},y_{j})$. (A5)

for all $i=1,...,n$. Writing Eqs. (A5) and (3) in a matrix form yields Eq. (5). Eq. (5) is formally similar to the Block Kriging equations [12], it focus on the estimation of the total number of disease cases in an area, it includes the additional coefficients *b_i_* to handle the biasedness of the sample (sentinel hospital records), and is expressed in a suitable discrete form to account for countable hospital distributions.

In light of Eqs. (3), (5), the 2^nd^ term in the right of Eq. (A4) can be written as

$2\sum_{i=1}^{N} \sum_{j=1}^{n} w_{j}C(y_{i},y_{j})=2\sum_{i=1}^{n} w_{i} [\sum_{j=1}^{n} C(y_{i},y_{j})+\mu b_{i}]=2\sum_{i=1}^{n} \sum_{j=1}^{n} w_{i}w_{j}C(y_{i},y_{j})+2\mu$,

in which case Eq. (A4) can be written as

, or

, (A5)

which is Eq. (6).
