# Supplementary material for: Area Disease Estimation Based on Sentinel Hospital Records
Source: PLoS One. 2011 Aug 23;6(8):e23428. doi: 10.1371/journal.pone.0023428 (PMC3160318; doi:10.1371/journal.pone.0023428)
Supplement: File S2 — A brief review of the ratio estimator. (DOC) [file pone.0023428.s002.doc]

**Supporting Information File 2. A brief review of the ratio estimator** [6]

The ratio estimator [6] seeks to correct the potential bias of sentinel hospitals records. The ratio estimator assumes that the proportion between the number of cases reported by all hospitals over that reported only by the sentinel hospitals is constant in time, in the sense that,

$$\sum_{i=1}^{N} y_{i}(t)/\sum_{i=1}^{n} y_{i}(t)=\sum_{i=1}^{N} y_{i}(t')/\sum_{i=1}^{n} y_{i}(t')$$

where $t$ denotes the estimation time and $t'$ is the same time but the previous year; *n* and *N* denote the numbers of sentinel hospitals and all hospitals in the area respectively. The number of disease cases reported by all hospitals in the area of interest, i.e. Eq. (1), can be estimated by the ratio estimator,

$y^{r}(t)=b_{n}\sum_{i=1}^{n} y_{i}(t)$, (A6)

where $b_{n}=\sum_{i=1}^{N} y_{i}(t')/\sum_{i=1}^{n} y_{i}(t')$. Here the *b_n_* is different from *b_i_* in Eq. (3), where .
