# Supplementary material for: Area Disease Estimation Based on Sentinel Hospital Records
Source: PLoS One. 2011 Aug 23;6(8):e23428. doi: 10.1371/journal.pone.0023428 (PMC3160318; doi:10.1371/journal.pone.0023428)
Supplement: File S3 — A brief review of the simple random estimator. (DOC) [file pone.0023428.s003.doc]

**Supporting Information File 3. A brief review of the simple random estimator** [8]

A simple random estimator is the simplest estimator of a health population attribute using the available data (sample), based on the assumption that the sample average is equal to the population average. This time, the number of cases reported by all hospitals in Eq. (1) is estimated by the simple random estimator

, (A7)

where, as before, $y_{i}$ is the number of cases reported by the *i*-th hospital; *n* and *N* denote the number of sentinel hospitals and all hospitals in the region, respectively. Eq. (A7) is a BLUE only if the sample is unbiased and independent. In particular, unbiasedness implies that $EY=Ey^{s}$, or , or $\sum_{i=1}^{N} y_{i}/\sum_{i=1}^{n} y_{i})=n/N$.
